# Evaluating the impacts of trap and lure costs and attractiveness on invasive insect trapping designs

**DOI:** 10.1038/s41598-026-53691-1

**Published:** 2026-05-20

**Authors:** Lauren Stutts, Barney P. Caton, Hui Fang, Nicholas C. Manoukis, Godshen Robert

**Affiliations:** 1 Center for Integrated Pest Management, North Carolina State University, Raleigh, NC 27606 USA; 2 United States Department of Agriculture, Animal and Plant Health Inspection Service, Plant Protection and Quarantine, 920 Main Campus Drive, Suite 200, Raleigh, NC 27606 USA; 3 European Biological Control Laboratory, United States Department of Agriculture – Agricultural Research Service, Montferrier-sur-Lez, 34980 France

**Keywords:** Invasive species, Surveillance trapping, Economics, Cost-benefit analysis, Ecology, Ecology, Zoology

## Abstract

We quantitatively examined the effects of trap and lure costs and attractiveness—which determines densities—on total insect survey costs. We modeled and compared costs for standard and reduced-density designs facilitated either by increased trap attractiveness, or by combining lures in traps. Survey costs were based on supply and replacement, and servicing distances and times. We quantified likelihoods of capture [*p*(Capture), survey efficacy] for each design in simulations. In a sensitivity analysis, total costs were most affected by trap density and survey area; trap-and-lure costs and durations were much less important. In a proof-of-concept example, we evaluated the impact of doubled attractiveness or tripled duration for *Anastrepha ludens* (Mexican fruit fly [Diptera: Tephritidae]). Increased attractiveness facilitated reducing traps by 61% and service distances by 58%, which decreased total costs by 44%. By contrast, increasing lure duration to 90 days in a 90-d survey only reduced costs by 6.5%. We then evaluated three case studies from published research. In two, though trap and lure prices at least doubled, total costs decreased by 42% or more because densities dropped by at least 59%. Moreover, *p*(Capture) at least doubled. In the third case study, combining three lures in traps reduced total costs by about 14% when attractiveness was unaffected, but when attractiveness declined, requiring greater densities, total costs increased 1.5 times. Incorporating traps with greater attractiveness usually reduced total costs and sometimes increased *p*(Capture). These examples demonstrate how to quantitatively assess trapping costs and survey efficacy to generate more optimal designs.

**Supplementary Information:**

The online version contains supplementary material available at 10.1038/s41598-026-53691-1.

## Introduction

Surveillance is a key biosecurity component that is used to detect, characterize, respond to, and monitor incursions of exotic plant pests^1,2^. For insect pests, using traps incorporating attractants to capture adults is a mainstay of surveillance^3^, particularly for fruit flies in the Tephritidae family^4,5^. Attractants, or lures, range from plant- or food-based compounds^6^ to sex pheromones and male lures^7^, while trap types have various glass, plastic, and paper designs^8^. Costs for the various trap-and-lure combinations can vary substantially. In addition to supply and lure replacement costs, insect trapping program costs also include operations, such as transportation costs and the labor for time spent traveling to, checking, and servicing traps e.g.,^9^. In that analysis, labor accounted for 69% of direct trapping costs, while supplies only accounted for 9%.

Survey efficacy and costs depend very strongly on trap-and-lure attractiveness, since this—along with survey size—determines the number of traps used. Survey designs, when available, usually proscribe the density for the corresponding trap-and-lure, and may also recommend the size of the area to cover. More attractive trap-and-lure combinations are highly desirable, for increasing *p*(Capture), for reducing costs, or for both, and much research goes to finding such combinations e.g.,^10–12^. Similarly, lure duration determines how many times replacements will be needed over the length of the survey e.g.,^13^, which also directly affects costs. Nevertheless, detailed consideration of survey costs is rare in most studies: In the trap and lure trials mentioned above, the authors referenced their costs but did not calculate and compare the effects of different options on total survey costs.

Our goal was to quantify how trap-and-lure attractiveness and costs interact with survey designs, total costs, and *p*(Capture), to better understand how to both optimize outcomes and economize. We built a detailed trapping costs model which accounted for supply and servicing costs, where servicing included both trap placement and required checks. We performed a sensitivity analysis to find the most important factors affecting total costs. In case studies, we used hypothetical trapping programs and real results from three research studies on trap attractiveness for different insect species. Every analysis was based on simulations with TrapGrid^14^ to determine *p*(Capture) for the trapping grids. We could then determine how using more attractive traps could reduce trapping densities while maintaining at least that *p*(Capture) level. Density reductions might also reduce service distances, providing further cost savings. In two cases we also evaluated the impact of enhanced lure duration. Combining the trapping density and service distance results with quotes and/or estimates of trap-and-lure costs allowed us to evaluate the overall impacts of improvements on total costs in detail.

First, as a proof of concept, we focused on the well-known example of quarantine trapping for *Anastrepha ludens* (Mexican fruit fly, Mexfly). Mexfly is a significant pest of several crops economically important to the United States, such as citrus^15^, and it is routinely managed in the Lower Rio Grande Valley in Texas after incursions and quarantines^16–18^.

Next, we evaluated three case studies from the literature, based on real trapping attractiveness results and cost estimates/quotes for the traps and lures. In the first, researchers tested adding attractants to pheromone traps for *Amyelois transitella* (Walker) (Lepidoptera: Pyralidae), commonly known as navel orangeworm (NOW)^19^. NOW is a pest of tree nuts such as almond, pistachio, and walnut, with annual impacts of millions of dollars (West Coast Nut 2023). The second case study was based on trapping research on the wasp-mimicking *Xylotrechus chinensis* (Chevrolat) (Coleoptera: Cerambycidae)^20^. *X. chinensis* is commonly (though not uniquely) known as tiger longicorn beetle (TLB). It is native to East Asia and is a pest of mulberry (*Morus* spp.) trees^21,22^. Kavallieratos et al.^20^ evaluated three attractant blends for trapping TLB and found consistent, stepwise increases in attractiveness. In the third case study, Stringer et al.^23^ tested fruit fly lures separately and in different combinations to evaluate the potential to use fewer traps. The lures tested were trimedlure (TML) for *Ceratitis capitata* (Wiedemann) [Mediterranean fruit fly, Medfly] (Diptera: Tephritidae), cuelure (CL) for *Bactrocera tryoni* (Froggatt) [Queensland fruit fly, Qfly] and *Zeugodacus* (*Bactrocera*) *cucurbitae* (Coquillett) [Melon fly], and methyl eugenol (ME) for *Bactrocera dorsalis* Hendel [Oriental fruit fly, OFF]. Attractiveness of combinations sometimes varied by location, but they identified three trapping design options which we evaluated here. In all examples, combining simulation results with estimated trapping costs allowed us to determine which survey specifications were the lowest cost and best performing given the various trap-and-lure combinations available, and better understand the drivers of survey cost differences.

## Materials and methods

### Calculating trapping survey costs

A starting equation for estimating total trapping survey costs (*C*_TOT_) is as follows^24^:

1 $${C_{{\mathrm{TOT}}}}={\text{ }}[{C_{\mathrm{T}}}+{\text{ }}({C_{\mathrm{L}}} \times {R_{\mathrm{L}}}){\text{ }}+{\text{ }}({C_{\mathrm{S}}} \times {R_{\mathrm{S}}})] \times {N_{\mathrm{T}}}$$

where *C*_T_ is the cost of each trap ($), *C*_L_ is the cost of the lure ($), *R*_L_ is the total number of lures needed during the survey, *C*_S_ is the salary cost of each servicing operation (per trap), *R*_S_ is the number of servicing operations, and *N*_T_ is the number of traps. [Trap density would equal *N*_T_ / (survey area).] One complication is that servicing operations include both trap checks (for captures) and lure replacements, or both, and each of those may take different amounts of time. In addition, surveyors spend time moving between traps, so the labor costs of traveling and any associated transportation costs are not accounted for in Eq. 1. Our calculations below assumed that all lure replacements would occur with trap checks, but this could be relaxed if needed. One related activity we did not include is handling and identification of captured insects, usually done in a laboratory, with associated salary costs for technicians.

**Fig. 1:**
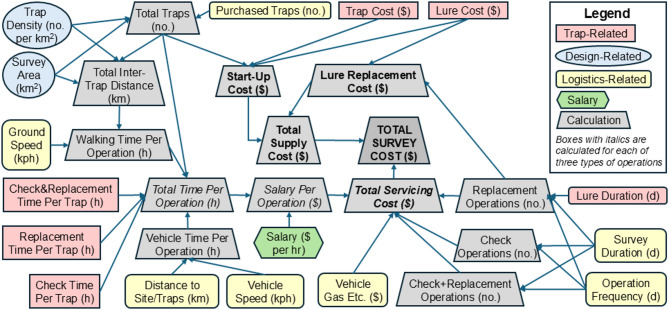
Model for calculating trapping survey costs based on the grid design and its effects on supply costs and operational logistics.

Our more complete model explicitly accounts for both supply (Eq. 1) and operations costs (Fig. 1). Operations consisted of checking and replacing traps and lures, traveling between all traps within survey sites, plus any vehicle costs (e.g., fuel). The model can account for variability in lure durations. We built this model as an MS Excel tool to quickly compare differences in trap and lure costs and any impacts on operations and the trapping grid (spatially and numerically).

*Constants.* We estimated surveys costs over 30 days based on initial trap placement (Day 1) followed by three additional weekly checks. All lure durations were at least 30 d, which meant zero replacements in the standard situation. We assumed that only lures would need replacement, not the traps. Service time included the travel time between traps, plus the time required to check or replace a lure or both for every trap; those explicitly depended on the frequencies of the activities (Fig. 1). Service times were 5 min (0.083 h) for each single activity, or 7 min (0.117 h) if both were done: these were realistic, arbitrary values. We manually calculated travel distances to every trap in the grid, but ignored travel to and from the survey sites, as that would be the same within each case study. Surveyor wage was always constant at $21 per hour, based on the average hourly rate (rounded to the nearest dollar) for a GS-7 federal employee in 2022^25^. Walking speed was assumed to be 4.8 kph (3 mph).

*Sensitivity analysis.* We used the basic model to evaluate the impacts of nine different factors on total cost as follows, with default values shown:

1. Check frequency–7 d.
2. Check time per trap – 0.083 h (5 min).
3. Check plus lure replacement time per trap – 0.117 h (7 min).
4. Lure cost – $4 per trap.
5. Lure duration – 30 d.
6. Salary – $21 per hour.
7. Survey area – 32.2 km^2^ (radius = 3.2 km).
8. Survey duration – 60 days.
9. Trap cost – $4 per trap.
10. Trap density – 13.9 traps per km^2^ (36 traps per mi^2^).
11. Walking speed – 4.8 km per h (3 mph).

The baseline survey had 448 traps, supply cost = $5,376, service cost = $12,711, and total cost = $18,087.

We evaluated sensitivity by increasing and decreasing each factor above by 50%, and calculating the proportional change in supply, service and total costs compared to the baseline. Final sensitivity values were the mean absolute proportional change.

*Noneconomic trap costs.* If using an improved trap-and-lure would save money at its estimated price, it could be useful to know the cost per unit (*C*_Unit_) at which it would no longer save money. We calculated this as follows:

2 $${C_{{\mathrm{Supp}}+}}={C_{{\mathrm{Tot}}0}} - {C_{{\mathrm{Serv}}+}}$$

3 $${C_{{\mathrm{Unit}}}}={C_{{\mathrm{Supp}}+}}/{N_+}$$

where *C*_Tot0_ is the total cost of the default survey, *C*_Serv+_ is the cost of servicing in the improved survey, and *C*_Supp+_ is the supply cost to be made up for total costs to be equivalent between the default and improved grids. In Eq. (3), *N*_+_ is the total number of improved units (traps or lures or both), and *C*_Unit_ is the trap-and-grid cost that would equalize C_Tot0_ and the total cost of the enhanced design (*C*_Tot+_). Note that *C*_Serv+_ is unaffected by trap or lure costs (Fig. 1). We also calculated the proportional increase in *C*_Unit_ over the default cost.

### TrapGrid simulations

We used TrapGrid (Version 2020.09.22) to estimate the likelihood of detection for standard and alternative survey designs^14^. We used the traditional algorithm of TrapGrid^26^, which calculates average capture probability, with default settings unless otherwise specified. Beside the grid of trap locations, the model requires number of simulations (*N*), number of insects per simulation (*n*), survey duration, the diffusion coefficient (*D*; m^2^ per day) for mobility, and trap attractiveness (1/λ). As in previous studies^14,27^ every simulation below used the same values of *N* = 50, *n* = 50, and duration = 30 d. *D* is the potential daily area explored by the organism^28^, with typical values for species ranging from near zero into the 10,000s of square meters per day^29^. Trap attractiveness, 1/λ, is the distance (m) between trap and target insect at which the insect has a 65% chance of being caught by a trap^14^. The trapping grids also depended on the species and the test (i.e., density reductions). Every simulation used the same random outbreak locations within the core square mile (see below).

Baseline parameter values for each case are shown in Table 1; some are cited below.

### Proof of concept: Mexfly trapping

To practically demonstrate the evaluation of improved attractiveness or duration of a trap-and-lure combination, we first used a hypothetical situation based on quarantine trapping for Mexfly. The USDA executes a Mexfly control program combining surveillance, quarantine protocols, and sterile insect releases to prevent its permanent establishment^30^. Current monitoring strategies for Mexfly involve a routine detection network, expanded delimitation surveys after detections, and intensive trapping during quarantines. The standard trap-and-lure is known for low attractiveness^18^ and has been a target for improvement for many years^6^.

**Table 1:** Values for key simulation model parameters and trapping cost factors.

Case study	Diffusion coefficient	Survey area	Trap attractiveness	Total traps	Costs	
	(m^2^ d^− 1^)	(km^2^)	(m)	(no.)	($)	
					Trap	Lure
Mexfly	265	169.2	**5.0**, 10.0	760	9.00	**1.00**, 2.00
Navel orangeworm	200,000	169.2	**15.0**, 19.0, 26.0	760	8.24	3.50, 4.16, 7.81^a^
Tiger longicorn beetle	458	18.3	**1.2**, 3.8, 6.9	3,522	8.24	**5.00**, 9.70, 18.55
Fruit flies						
Medfly	1,240	201.1	**10.0**, 2.45	1,268	1.93^b^	1.70
Melon fly/Qfly	110 / 1,600	201.1	**27.0** ^c^	1,268	1.93	3.43
OFF	60,000	201.1	**36.0**	160	1.93	1.40

Results are for the proof-of-concept example (Mexfly) and three case studies, showing the diffusion coefficient, survey size, trap attractiveness (1/λ), total traps in the standard grid, and trap and lure costs. Among multiple values, bold values indicate the default for tests. See text for references.

^a^ In the following order: PPO, pheromone, and PPO plus pheromone.

^b^ Includes $0.20 for the sticky insert.

^c^ We used the empirical value for melon fly^31^ for both species, based on similar capture rates in Stringer et al. (2019).

*Trapping factors and costs.* The Mexfly trap is a McPhail baited with the 2-component lure, which consists of putrescine and ammonium acetate^32^. It has a useful duration of 30 days. We set a default value of 1/λ = 5 m. Based on this, doubled attractiveness simply means 1/λ = 10 m. The prices were $9 per Multilure^®^ trap, and $1 per dose of the standard lure (Better World Manufacturing Inc., Fresno, CA, USA; https://www.betterworldus.com) (Table 1). The hypothetical enhanced lure had a price of $2. We first evaluated costs with the default survey and lure durations (above). In a second test, we adopted a 90-d survey duration and then compared total costs with the standard lure duration (30 d) and with an improved lure duration of 90 d (i.e., zero replacements) but with no improvement in attractiveness. We assumed the cost of the lure doubled, with no change in the trap cost (Table 1).

*Diffusion coefficient.* The value of *D* for Mexfly has been estimated at 265 m^2^ per day ^46^, ^47^(Table 1).

**Fig. 2:**
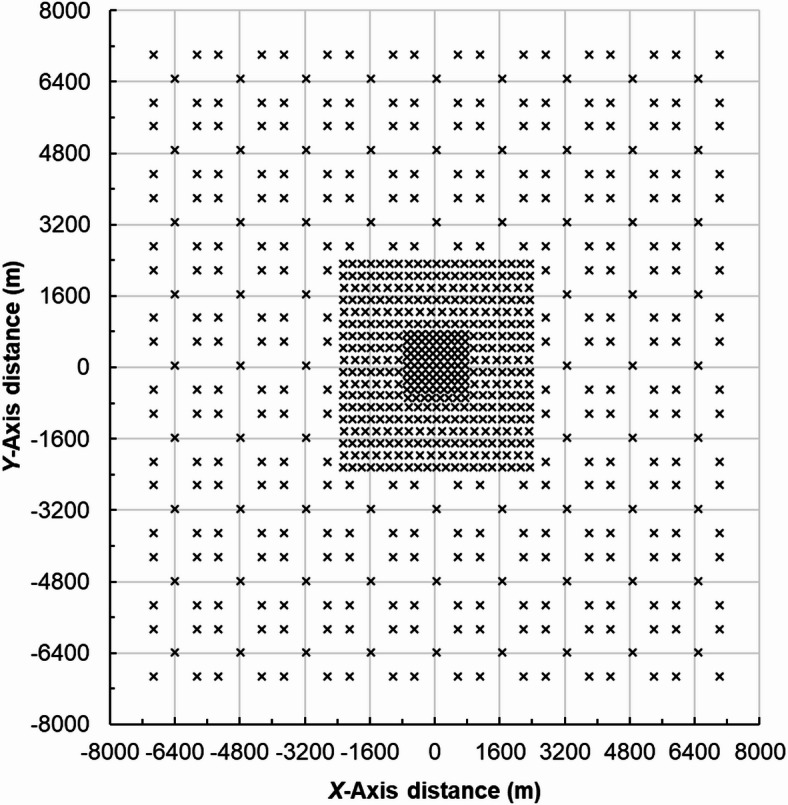
Locations of 760 traps (Xs) in the standard grid for Mexfly, with square-mile cells (2.6 km^2^). The densities (traps per km^2^) are as follows: core area = 30.9 [80 traps per mi^2^]; second band = 15.4 [40 traps per sq. mi] and all other bands = 1.9 [5 traps per sq. mi].

**Survey design**. The standard quarantine trapping grid for Mexfly has 760 traps in a 25 square mile square (When implemented in the field, survey managers typically round off the corners of the square.) (Fig. 2), arranged in concentric bands of 30.9, 15.4, 1.9, 1.9, and 1.9 traps per km^2^ (80, 40, 5, 5, and 5 traps/mi^2^)^8^. In the TrapGrid model simulation this grid had a likelihood of capture of 0.233. To simplify the creation of grids we usually tested reduced densities in each band that were squares (36 traps per mi^2^, 25, 16, etc.), setting a minimum trap density of 0.8 traps per km^2^ (2 traps per mi^2^). Each grid was then simulated in TrapGrid to determine the likelihood of capture. Varying densities in each band complicated the process, but we stopped when we had minimized total trap number while approximately matching the baseline likelihood of capture.

### Case studies

#### Navel orangeworm

**Trapping results**. Surveillance for NOW employs pheromone-baited delta (wing) traps^33^. Burks et al.^19^ recently tested whether adding phenyl propionate (PPO) to the pheromone would increase capture rates. In almonds, compared to PPO-only lures, captures increased 27% with the pheromone, and increased 77% with PPO + pheromone.

**Attractiveness**. Mark-release-recapture data to establish accurate attractiveness values was unavailable. Based on capture rates, PPO-only traps had only moderate attractiveness at best, so we assumed 1/λ = 15 m as a baseline. Given the capture results from the study, the pheromone-only trap had 1/λ = 19.0 m, and the PPO+pheromone trap had 1/λ = 26.5 m for (Table 1). While the baseline estimate may not be accurate, the values were realistic (as were the associated trap densities, below), and as a relative measure would adequately represent the survey outcomes.

**Diffusion coefficient**. NOW is reported to be highly mobile^34–36^, so we used *D* of 200,000 m^2^ per day (Table 1).

**Trap and lure costs**. All lures had durations of six weeks^19^. Exact costs for these lures were unavailable, but based on comparable, commercially available lures, we assumed $3.50 per lure for PPO, $4.16 (19% greater) for each pheromone-only lure, and $7.81 for the PPO+pheromone lure (105% greater; Great Lakes IPM™, Vestaburg, MI; https://www.greatlakesipm.com) (Table 1). The cost of the delta trap was $4.60 (Evergreen Growers Supply, LLC, Clackamas, OR; https://www.evergreengrowers.com).

**Fig. 3:**
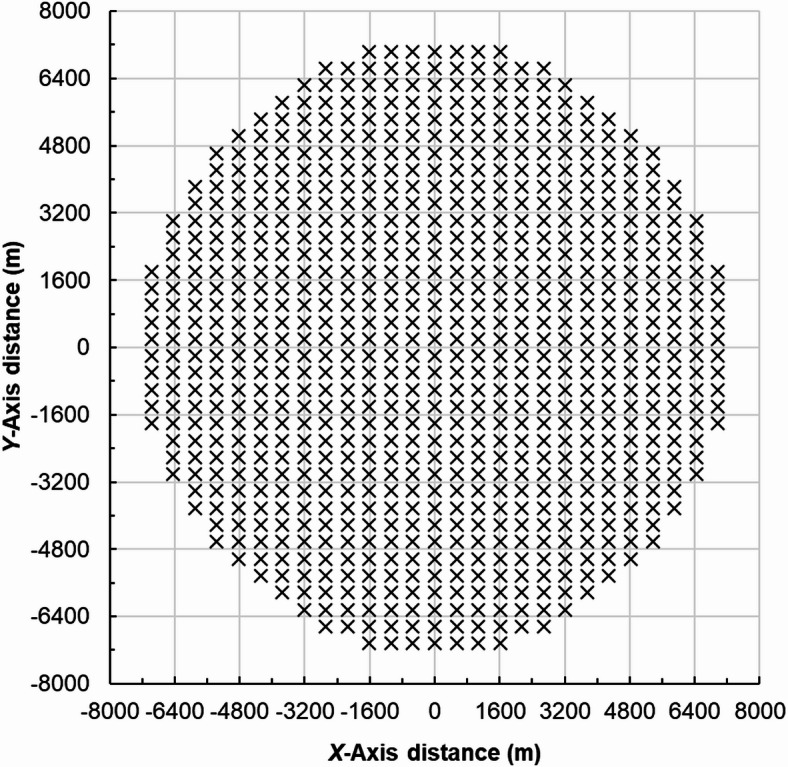
Standard trap locations (Xs) for the navel orangeworm case study. With a density of 4.6 traps per km² (12 traps per mi²) in square-mile cells (2.6 km^2^), there are 760 total traps.

**Survey design**. Based on its high dispersal ability, we set a survey radius of 7.25 km (area = 165.1 km^2^; Fig. 3). The standard trap density for pheromone traps for NOW is about 5 traps per km^2^^37^, so our default rate was 4.6 traps per km (12 per mi^2^). The survey area had 760 total traps at that density (Table 1).

#### Tiger longicorn beetle

**Trapping results**. Kavalliertos et al.^20^ reported captures of TLB with three attractants in multi-funnel traps. The attractants were mixes of seven chemicals and prionic acid + ethanol (lure 1), plus α-pinene (lure 2), and also plus ipsenol (lure 3). The reported mean capture numbers (adults per week per trap) were 0.31 for lure 1, 0.91 for lure 2, and 1.66 for lure 3. Hence, captures increased by a factor of 2.9 for lure 2, and by 5.3 for lure 3.

**Attractiveness**. We used TGL-Lambda^38^ to estimate the baseline 1/λ value for lure 1. Based on the assumption that the capture rate for lure 1 represented an 0.98 escape probability, we set 1/λ = 1.215 m. The attractiveness values for the other two attractants were then scaled proportionally based on the differences in the reported capture rates above^20^. For lure 2, 1/λ = 3.54 m, and for lure 3, 1/λ = 6.45 m (Table 1). Although the accuracy here is uncertain, the comparative values should reasonably represent relative effects on *p*(Capture).

**Diffusion coefficient**. Based on a low to moderate mean annual spread rate for TLB of about 50 km^2^^39^, we set *D* = 458 m^2^ per day (Table 1).

**Trap and lure costs**. The estimated price for the multifunnel trap was $8.24 (Forestry Distributing, Longmont, Colorado; https://www.forestrydistributing.com) (Table 1). We had no information on the costs of lure 1^20^, so we assumed a base cost of $5 per vial. Based on an author suggestion (Kavallieratos, personal communication), the corresponding costs would be $9.70 per vial for lure 2, and $18.55 per vial of lure 3. We assumed lure durations were greater than the 30-day survey duration.

**Fig. 4:**
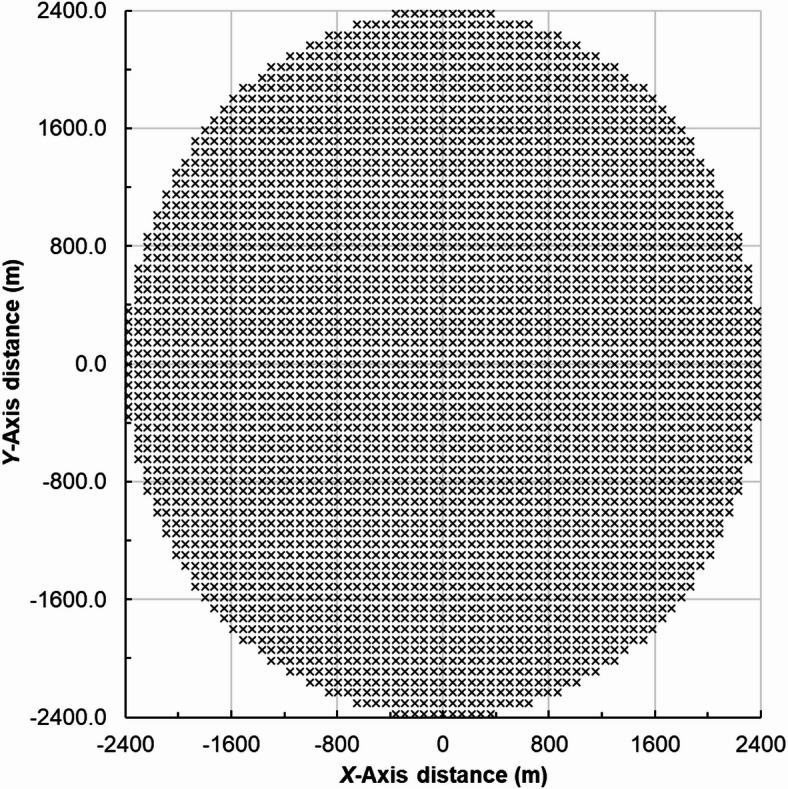
Trap locations (Xs) in the standard grid design for the tiger longicorn beetle. At a density of 241.3 traps per km² (625 traps per mi²), this design has 3,522 total traps.

**Survey design**. Given the low mobility rate for TLB, the survey radius was only 2.4 km (1.5 mi), which gave an area of 18.1 km^2^. Given low attractiveness of lure 1 (above), we set a default trap density of 241.3 traps per km^2^ (625 traps per mi^2^), which gave 3,521 total traps (Table 1; Fig. 4).

#### Fruit fly lure combinations

The three major male lures used in surveillance, monitoring and control of tephritid fruit flies of economic importance are cuelure (CL), methyl eugenol (ME), and trimedlure (TML)^7^. Each lure targets different species. For areas with multiple fruit fly species present, traps baited with two or more of the male lures have been studied^40–42^ as a way to possibly reduce supply and service costs. Capture rates of the combined lures are sometimes inconsistent, however, as demonstrated by Stringer et al.^23^. We used that example for our case study.

The three trapping design options identified by Stringer et al.^23^ for detecting the target fruit fly species were as follows:

- Option 1: Three lures in separate traps at standard densities for each (Table 2)^43^.
- Option 2: ME is separate at its standard density, but TML and CL are combined in traps at their (equal) standard density.
- Three lures in the same trap, at 6.25 traps per km^2^ in Hawaii (option 3a), or at 19.8 traps per km^2^ in Western Australia (option 3b), because TML was negatively affected there by the combination lures.

**Trapping results**. In Western Australia trials, mean trap catch for Medfly with TML dropped by over 75% in the trap with all three lures, from 27.9 to 6.8 (Figure 3B in Stringer et al.^23^). In trials in Hawaii (Figure 1 in Stringer et al. ^23^) the only significant difference in capture rate reported was for OFF with ME, with a decrease when all three lures were combined. The mean trap catch decreased by 68% from about 92 to 29 adults.

**Attractiveness**. For CL, the value of 1/λ for melon fly was estimated to be 27 m^31^ and that never changed when combined with others (Table 2). We have no estimate for Qfly with CL in Australia, but assumed it was the same value as for melon fly based on similar capture rates in Hawaii (Fig. 1 in Stringer et al.^23^). We have good evidence that the attractiveness of Medfly to TML is equal to 10 m^44^ (Table 2). For combined traps with TML in Western Australia, 1/λ would drop to 2.45 m. The attractiveness of OFF to ME is very high, at 36 m^45^, but in Hawaii in option 3a, 1/λ for ME became only 11.3 m.

**Table 2:** Key simulation model parameters.

Organism	Attractiveness (1/λ)				Trap density (no. per km^2^)			
	Option 1	Option 2	Option 3		Option 1	Option 2	Option 3	
			HI	WA			HI	WA
Medfly	10.0	10.0	10.0	2.45	6.2	6.2	6.2	18.9
Melon fly/Qfly^a^	27.0	27.0	27.0	27.0	6.2			
OFF	36.0	36.0	11.3	36.0	0.8	0.8		

Values are for the fruit fly lure combinations case study, showing trap attractiveness and trap densities in different options.

^a^ The three species being trapped were Medfly, OFF, and either melon fly or Qfly.

**Diffusion coefficients**. For Medfly, *D* = 1,240 m^2^ per day, and for OFF, *D* = 60,000 m^2^ per day^46,47^ (Table 1). For the other two species we calculated estimates using collated data in Weldon et al.^47^, which gave *D* = 110 m^2^ per day for melon fly, and *D* = 1,600 m^2^ per day for Qfly.

**Trap and lure costs**. The study only used Lynfield traps ^23^ but we used the Jackson trap (plastic) for cost comparisons here, as those are more widely used in the US. They cost $1.93 with the sticky insert (Better World Manufacturing Inc.) (Table 1). [Note: inserts are a relatively minimal cost at about $0.20 so we included replacements in calculations.] The price for a TML lure for *C. capitata* was $1.70, while the price for ME for OFF was $1.40 (Better World Manufacturing Inc.). Finally, the cost of a CL lure for melon fly and Qfly was $3.43 (Evergreen Growers Supply, LLC).

**Fig. 5:**
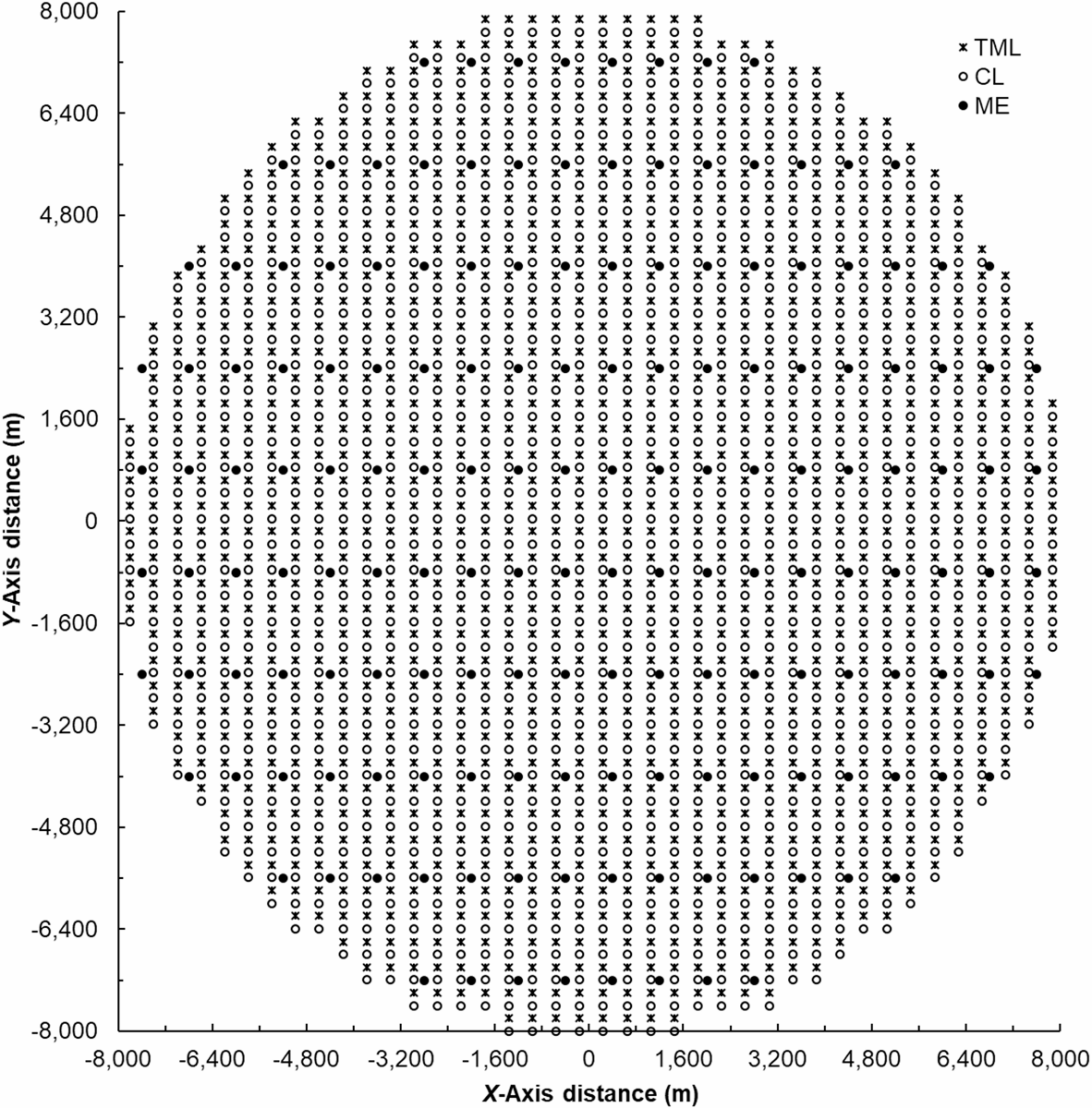
Trap locations in the first grid design option for fruit flies. This includes traps at a density of 6.25 traps per km^2^ (16 traps per mi^2^) with TML and CL, and traps at 0.8 per km^2^ with ME. This design has 2,736 total traps.

**Survey design**. The grid area was 201.1 km^2^ (radius = 8 km). The standard densities were 6.25 traps per km^2^ for TML and CL traps, and 0.8 traps per km^2^ for the ME trap (Table 2)^43^. In option 1 these would be placed together in the grid (Fig. 5), maintaining TML and CL traps in the same columns to minimize the service distance. In option 2, TML and CL are in the same traps, and *p*(Capture) should not change from option 1. In option 3a all lures are in the same traps at the standard density for TML and CL traps, so *p*(Capture) should not change for them, but would for ME because of the density increase. In option 3b, for Western Australia, the trap density for all lures would increase to 18.9 traps per km^2^, probably strongly affecting all *p*(Capture) values. Option 1 had 2,696 total traps, option 2 had 1,428 traps, option 3a (HI) had 1,268 traps, and option 3b (WA) had 3,852 traps (Table 1).

## Results

### Trapping cost model sensitivity

Trap density and survey area had the greatest impact on trapping costs (Table 3), which was reasonable given that both parameters directly affect both supplies and servicing operations (Fig. 1). Trap density sensitivity was affected equally by supply and servicing costs, while survey area sensitivity depended less on servicing costs (Table 3). Salary and survey duration sensitivities were also substantial. A gap existed before the next three factors, which only affected servicing costs. The sensitivity of costs to lure duration and trap and lure costs were moderate for supply costs but low overall.

**Table 3:** The sensitivities of nine trapping cost model factors compared to baseline costs.

Rank (Total)	Factor	Mean absolute proportional change		
		Total cost	Supply cost	Servicing cost
1	Trap density	0.499	0.499	0.499
2	Survey area	0.427	0.499	0.397
3	Salary	0.351	0.000	0.500
4	Survey duration	0.311	0.333	0.302
5	Check frequency	0.216	0.000	0.307
6	Walking speed	0.198	0.000	0.281
7	Trap check time	0.173	0.000	0.246
8	Lure duration	0.117	0.333	0.025
9	Lure cost	0.099	0.333	0.000
10	Trap cost	0.050	0.167	0.000
11	Trap check + lure replace time	0.030	0.000	0.043

Factors are ranked by impacts on total cost.

### Proof of concept: Mexfly trapping

**Fig. 6:**
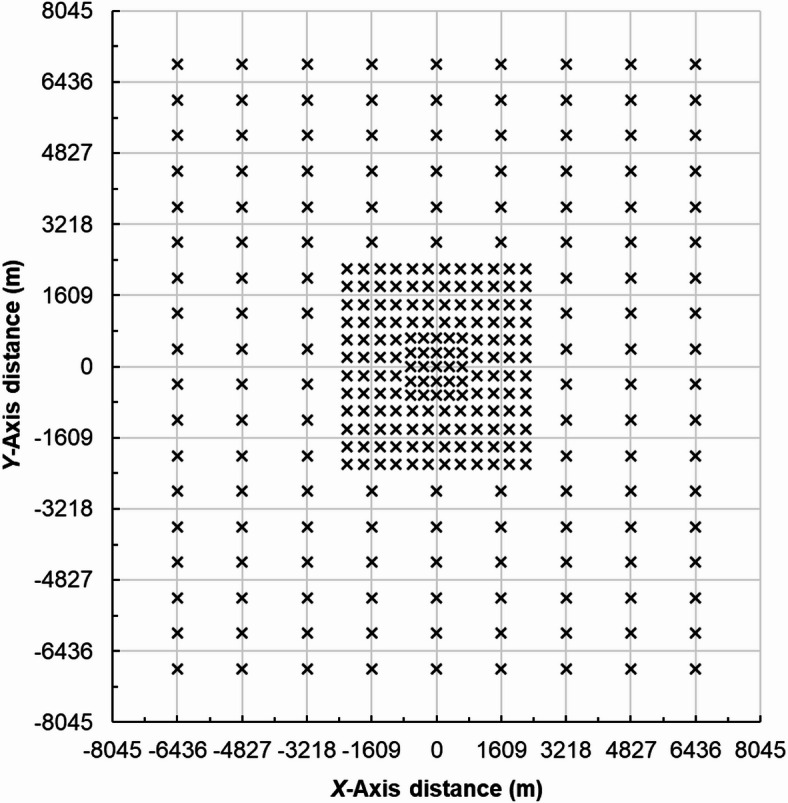
Trapping grid for Mexfly. Locations of 297 traps (Xs) are shown after incorporating a trap-and-lure combination with doubled attractiveness (and price).

The standard Mexfly grid had *p*(Capture) = 0.226. We found that a grid with the improved trap and lure and densities (traps per km^2^) of only 9.7 [band 1], 6.2 [band 2], and 0.8 [bands 3–5] slightly exceeded that, at 0.262 (Table 4; Fig. 6). That grid had 297 total traps, which was 61% less than the standard grid. This led to a 50.3% reduction in total trapping costs, bolstered by a 57% reduction in supply costs (Table 5). Servicing costs did not decrease as much because the distance only decreased by 37% (compare Figs. 2 and 6).

In the 90-d survey with tripled lure duration, the total cost only declined from $34,056 to $32,232 (5.4% decrease; Table 5). Supply costs decreased by 8.3% and servicing costs decreased by 4.3%.

**Table 4:** Trapping metrics.

Species	Area	*p*(Capture)		Density (traps/km^2^)		Traps (no.) [%]		Service Distance (km) [%]	
	(km^2^)	Std.	Red.	Std.	Red.	Std.	Red.	Std.	Red.
Mexfly	209.7	0.226	0.262	31-15-2-2-2	10-6-1-1-1	760	297 [-61]	1590.3	1002.9 [-37]
[Band 1]	2.6	—	—	30.9	9.7	80	25	—	—
[Band 2]	20.7	—	—	15.4	6.2	320	96	—	—
[Band 3]	41.4	—	—	1.9	0.8	80	56	—	—
[Band 4]	62.1	—	—	1.9	0.8	120	40	—	—
[Band 5]	82.8	—	—	1.9	0.8	160	80	—	—
Navel orangeworm^a^	162.9	0.43	0.42	4.6	2.3	760	382 [-50]	1590.3	1126.2 [-29]
Tiger longicorn beetle									
Lure 1 vs. Lure 3	18.3	0.094	0.081	241.3	6.2	3521	112 [-97]	1135.9	203.4 [-82]
* p*(Capture) ≈ 0.4^b^	18.3	0.38	0.39	98.8	31.3	1834	587 [-68]	828.2	459.4 [-45]
Fruit fly combinations									
Option 1	201.1	0.33	—	6.2/6.2/0.8^c^	—	2,696	—	17,500	—
Option 2	201.1	0.33	—	6.2/0.8^d^	—	1,428	—	10,075	—
Option 3 (HI)^e^	201.1	0.30	—	6.2	—	1,268	—	7,425	—
Option 3 (W.A.)	201.1	0.68	—	18.9	—	3,852	—	12,920	—

These are given for the proof-of-concept example (Mexfly) and two case studies for the standard grid (Std.) and the reduced-density grid (Red.) with an improved trap-and-lure combination. Metrics include survey areas, simulated likelihood of capture [*p*(Capture)], trap densities, numbers of traps, and baseline service distances (equal to the per-operation distance).

^a^ Traps with pheromone-only or PPO+pheromone.

^b^ Lure 2 vs. Lure 3.

^c^ Densities for Medfly / Melon fly or Qfly / OFF, in order.

^d^ Densities for Medlfy *and* Melon fly or Qfly (combined) / OFF, in order.

^e^ Densities are different depending on whether the trapping would be done in Hawaii or Western Australia.

**Table 5:** Supply, servicing, and total trapping costs.

Example	Situation	Supply cost ($)		Servicing cost ($)		Total cost ($)		
		Std.	Red. [% decrease]	Std.	Red. [% decrease]	Std.	Red.	Percent change^a^
Mexfly	Doubled attractiveness	$7,600	$3,267 [57.0]	$10,948	$5,947 [45.7]	$18,548	$9,214	-50.3
	Triple duration (90 d)	$9,120	$8,360 [8.3]	$24,936	$23,872 [4.3]	$34,056	$32,232	-5.4
Navel orangeworm	Phero. vs. PPO+Phero.	$6,658	$4,741 [28.8]	$10,948	$6,932 [36.7]	$17,605	$11,673	-33.7
Tiger longicorn	Lure 1 vs. Lure 3	$46,618	$3,000 [93.7]	$23,455	$1,478 [93.7]	$70,073	$4,478	-93.6
beetle	*p*(Capture) ≈ 0.4^b^	$32,902	$15,726 [52.2]	$13,252	$5,092 [61.6]	$46,154	$20,817	-54.9
Fruit	Option 1	$11,932	—	$74,066	—	$85,998	—	—
Fly	Option 2	$9,485	—	$74,066	—	$83,551	—	-2.8
Combinations	Option 3 (HI)	$10,727	—	$59,112	—	$69,840	—	-18.8
	Option 3 (WA)	$32,588	—	$137,415	—	$170,003	—	97.7

These are given with proportional change (in square brackets] for four case studies of insect trapping surveys in different situations with a standard grid (Std.) and a reduced-density grid (Red.) with a more attractive trap-and-lure combination, if applicable. Also shown are percentages of total costs for the reduced-density grids.

^a^ Relative to the standard total for Mexfly, NOW, and TLB, and the total of Option 1 for fruit flies.

^b^ Lure 2 vs. Lure 3.

### Case studies

#### Navel orangeworm

**Fig. 7:**
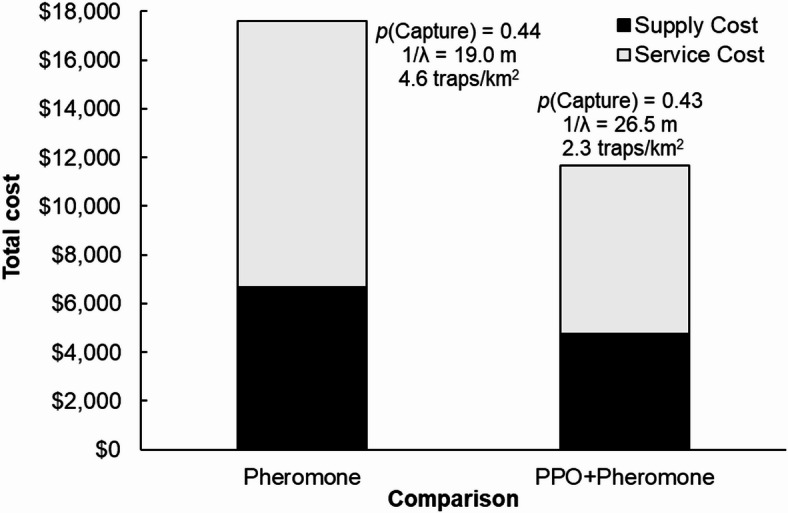
Total costs for trap-and-lure combinations for navel orangeworm. Displayed are options with different trap attractiveness (1/λ values; see text), with supply and servicing cost portions and the likelihoods of detection (*p* values) for each.

The default grid with 760 pheromone-only traps (Fig. 3) had *p*(Capture) = 0.44 (Fig. 7). Using PPO+pheromone yielded a grid with about half as many traps (Supp. Fig. S2) and similar *p*(Capture) of 0.43, but the total cost was almost 34% less (Table 5; Fig. 7). Almost 70% of the reduction came from lower servicing costs. Additionally, the PPO+pheromone lures are more effective around mating disruption efforts, which is a common strategy for reducing NOW populations^19^.

#### Tiger longicorn beetle

**Fig. 8:**
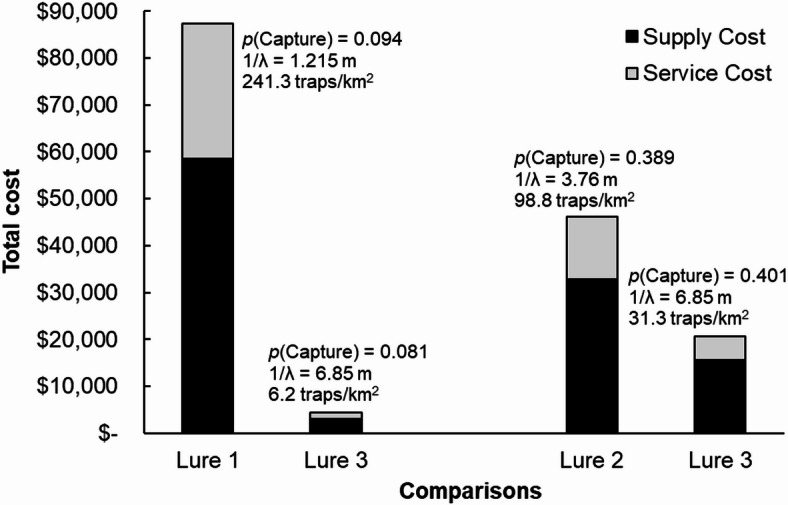
Total costs for trap-and-lure combinations for tiger longicorn beetle. Displayed are options with different trap attractiveness values (1/λ; see text), showing supply and servicing cost portions and the *p*(Capture) for each.

We first compared lure 1 with lure 3. Despite the default grid with lure 1 having 3,521 traps (Fig. 4), *p*(Capture) was only 0.094 because of its low attractiveness (Table 4). Lure 3 achieved *p*(Capture) = 0.081 with a trap density of only 6.2 traps per km^2^; the grid had only 112 traps (Fig. S3A). The 97% reduction in traps facilitated a 93% reduction in total costs (Table 5; Fig. 8). The reduction was about split between supply and servicing.

Secondly, to achieve greater *p*(Capture), lure 2 at a density of 98.8 traps per km^2^ gave a grid with 1,834 traps (Fig. S3B) and *p*(Capture) = 0.389 (Table 4). With lure 3, a grid with only 587 traps (31.3 traps per km^2^) (Fig. S3C) had *p*(Capture) = 0.39. Compared to the grid with lure 2, the grid with lure 3 cost about 55% less (Table 5; Fig. 8), and 68% of the reduction came from supply costs.

#### Fruit fly lure combinations

**Fig. 9:**
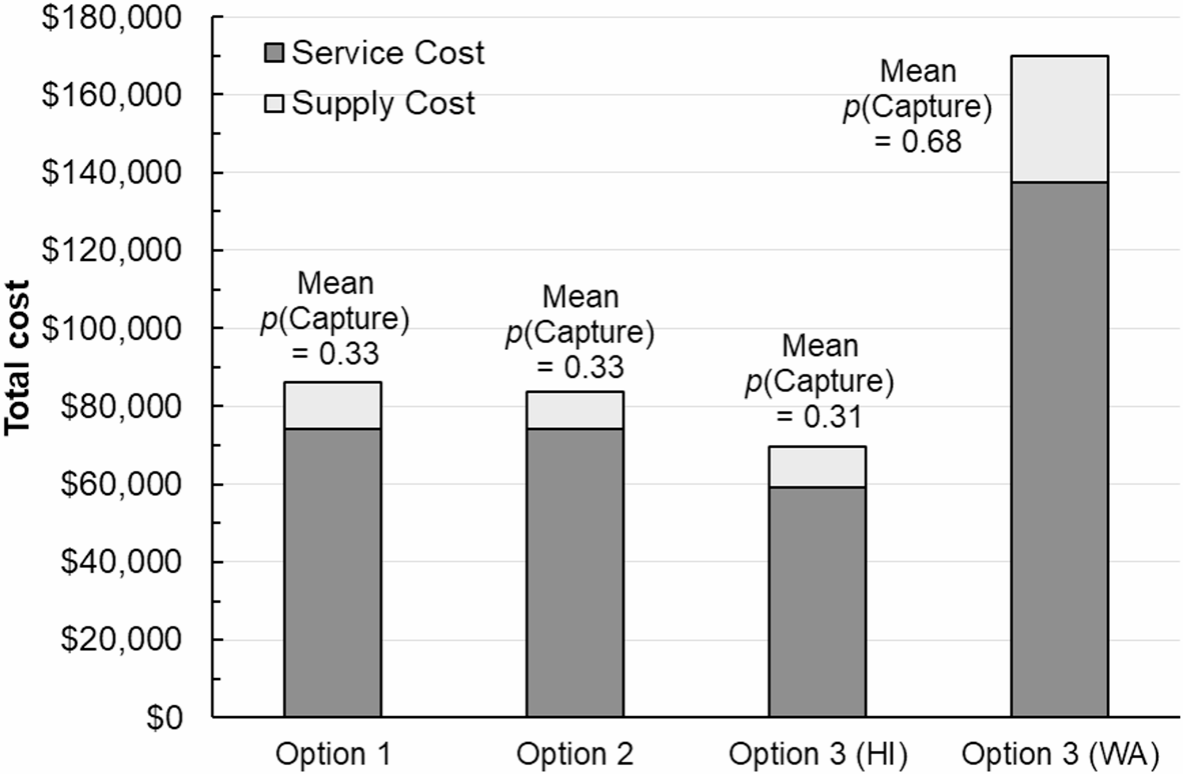
Total costs for trap-and-lure options for fruit flies. Displayed are different combinations of lures in traps (see text), showing supply and servicing cost portions and the mean *p*(Capture) for each.

The default situation in this case study was each lure with its own trap and density, which cost $85,998, of which 86% were service costs (Table 5; Fig. 9). Option 2, with TML and CL combined into traps, reduced costs by only about 3%, because the grid for option 1 already placed CL and TML traps in such a way as to minimize service costs (Fig. 5). With option 3a in Hawaii, the cost dropped by almost 19% (Table 5; Fig. 9), because this integrated ME into the basic service path, reducing the distance. Mean *p*(Capture) values for these three options were very similar (Table 6; Fig. 9). Total costs almost doubled for Option 3b in Western Australia (Table 5; Fig. 9), because the greater trap densities needed there to maintain TML effectiveness for Medfly substantially increased both supply and servicing costs. Despite the increase in trap density to mitigate lower attractiveness of TML, *p*(Capture) for Medfly was only 0.04.

**Table 6:** Values for *p*(Capture) for trapping grids.

Fruit fly	*p*(Capture)			
	Option 1	Option 2	Option 3	
			HI	WA
Medfly	0.187	0.187	0.187	0.039
Melon fly	0.507	0.507	0.507	
Qfly				0.994
OFF	0.296	0.296	0.238	1.000
Mean	0.330	0.330	0.310	0.678

Columns represent average capture probability for four fruit flies with three combinations of different lures with varying levels of attractiveness into traps (see text) for Medfly and OFF. For option 3 values are melon fly (HI) or Qfly (WA) plus Medfly and OFF.

## Discussion

### Survey efficacy and designs

We demonstrated two ways that estimating *p*(Capture) informs—and perhaps should underpin—the trapping survey design process. First, it can verify or refute expectations about survey efficacy. For example, in the TLB case study, lure 3 had the greatest attractiveness but had *p*(Capture) of only 0.08 at a density of 6.2 traps per km^2^ (Fig. 8). That density would probably only be chosen if limited by the budget. Spending $16K more to raise *p*(Capture) to 0.40 would bring an excellent return on investment. Second, working from a known, target level of *p*(Capture) facilitates setting a trapping density that meets that threshold while minimizing costs. The NOW case study demonstrated using a more attractive lure with a lower density without compromising *p*(Capture) (Fig. 7). Quickly comparing costs while accounting for potential survey performance seems desirable and beneficial.

Survey managers likely understand that greater attractiveness facilitates using lower trap densities, e.g.,^3^. Real-life evidence for this is that densities for trapping OFF with highly attractive ME lures are typically > 85% smaller than densities for trapping Medfly^8,43^, cf.^13^. However, that idea has rarely been stated explicitly or quantified in published studies. That was true for the case studies reviewed here, although Stringer et al.^3^ suggested a density *increase* required by poorer lure performance in combinations in that study. The development of most published survey plans—meaning trapping grids and density recommendations e.g.,^5,8^—has probably been subjective, based on expert knowledge of trap-and-lure attractiveness (to determine trap density) and insect mobility (to define the size of the survey area)^27^. Those specifications were likely balanced against total survey costs and potential budget limitations. Decreasing densities while moving away from the epicenter in the Medfly delimitation survey^8^ may be an example of this. Developers likely also had some intuitive estimate of a target level for *p*(Capture) for each design to help determine trap densities.

Tools such as TrapGrid have made this estimation process much more objective. Still, while the model is well-cited e.g.,^48–50^, it has generally seen limited application beyond a study by van Klinken et al.^51^, an online implementation^52^, and work by our group^27,44,53^. Without simulations, the nonlinear relationship between trap density, attractiveness, and *p*(Capture) can be approximated by interpolating Fig. 6 in Manoukis et al.^14^.

We used TrapGrid with a diffusion process for dispersal and calculated average escape probability over time^14^. This is appropriate for comparing alternative network designs, while a later version that enables calculating the likelihood of capturing one or more organisms is more relevant to low prevalence situations^26^. The newer model was used with an alternative random-walk dispersal process to investigate using trapping to verify site-free pest status^51^. That dispersal method works particularly well for a smaller number of individual insects. The original version worked well for our purposes and with non-scant insect populations, especially considering that the differences between *p*(Capture) values mattered much more than the accuracy of single values.

Some researchers have tested reducing trapping survey costs with lower-priced traps and lures rather than more attractive ones; often these are natural products or locally sourced^24,54,55^. Assuming no changes in trap densities, this tactic could be effective at reducing supply costs, but survey efficacy might suffer. Moreover, because total survey costs are relatively insensitive to trap costs (Table 2), only substantial decreases in trap or lure prices are likely to be very worthwhile. Similarly, in the short-duration surveys investigated here, improved lure duration was unlikely to give a substantial return on investment (see Mexfly example above). That tactic would probably be more worthwhile with longer, large-area surveys with many thousands of units^13^.

### Trapping cost factors

Estimating trapping survey costs quantitatively and in detail can be enlightening but has rarely been incorporated into relevant studies on potential combinations of traps-and-lures; de la Mora-Castañeda et al.^56^ is a notable exception. For example, Stringer et al.^23^ conjectured that when combining all lures in Western Australia, reduced servicing costs would offset the increased supply costs. However, our evaluation indicated costs would almost double because *both* supply and servicing costs would increase, which was an example of gaining insight by calculating and analyzing survey costs. Examples of publications in which costs could have been usefully calculated and compared include the case studies here, plus Agrafioti et al.^57^, Brockerhoff et al.^58^, and Lasa and Cruz^12^. One reason researchers may not do this might be a lack of standard survey design plans for the target insect (e.g., TLB). We think formally calculating detailed survey costs in cost-related trapping research would be good practice.

The three examples here demonstrated that incorporating traps-and-lures with improved attractiveness into reduced-density trapping grids saved from 34 to over 90% in total costs (Table 5). Generally, this type of savings will depend on the level of enhancement—which affects how much trap densities can be reduced—and relative trap and lure costs for the original and new versions. Alternately, instead of minimizing costs, some managers could use enhancements to improve *p*(Capture) for that trapping grid (e.g., Fig. 9). Improved capture probability might be particularly helpful for high-risk target pests, to avoid problematic and costly non-detections.

The third case study tested whether fruit fly lures could be combined into fewer traps to save survey costs. When co-placement had no or moderate effects on the attractiveness of lures, as in the Hawaii trials^23^, combining lures into traps saved money, but only up to 19% of the costs (Table 5). The switch to option 3 primarily reduced service costs. We also calculated the total cost if the density of ME lures (160 traps) was maintained despite the higher density of traps for TML and CL, but that only saved an additional 2% (not shown).

When greatly increased densities were needed to offset apparent interference on the TML lure in Western Australia^23^, the number of traps and lures needed increased well beyond the baseline number of *separate* traps, and service costs increased by 86% (Table 4). Offsetting costs may have seemed possible but quantification projected a large increase. In addition, because of the significant drop in attractiveness, increasing the trap density substantially still did not achieve even the baseline rate of *p*(Capture) for Medfly, which was 0.19 with separate traps-and-lures (option 1) and only 0.04 with combined lures (Table 5). Option 3b already seems too expensive to operate, so adding perhaps five times *more* traps (0.19 / 0.039 = 4.8) to try to raise the *p*(Capture) level seems undesirable.

We previously showed how “right-sizing” surveys based on adjusting the coverage area to insect mobility can save substantial amounts of money^27,44,46^. However, results here highlighted that savings did not solely depend on reducing survey areas. Reducing trap densities obviously lowered supply costs, but also always decreased servicing distances and costs (Table 4). Trap and lure costs, and lure durations, on the other hand, had minimal impacts on total survey costs (Table 3), because most costs came from servicing (Table 5), and only lure duration affected servicing costs. Although some studies have targeted increased lure durations to reduce costs e.g.,^10,13^, our results indicated that the impacts were small. Still, even marginal improvements may be desirable and meaningful to survey managers.

### Implications for trapping survey designs

In these case studies improved traps-and-lures were nearly always worth using if they facilitated using significantly fewer total traps. Although we cannot prove that is always the case, the estimated *non*economic costs of improved traps-and-lures (Eq. 2) here were very high. The break-even point for Mexfly would require the improved trap-and-lure cost to increase to almost $50, or about five times greater than the default cost (Table 7). For the NOW case study, the noneconomic cost of the improved lure was about 7 or 10 times greater than the original cost, depending on which lure was the default. With TLB, lure 3 is so much better than lure 1 that its price would have to be about 58 times greater to not be worth using (Table 7). The noneconomic cost difference compared to lure 2 was much smaller but still over a factor of 5.

Using better trapping surveys should improve pest management outcomes. Nguyen et al.^59^ pointed out that delayed detections from surveillance programs can increase control costs. They addressed the impacts of time-to-detection on pest eradication potential and included pest damages in surveillance costs. By contrast, we considered surveillance to be separate from control and did not evaluate time to detection from a design perspective. However, other studies have conclusively shown that trap density and attractiveness are critical for determining both *p*(Capture)^27^ and time to detection^51^, so higher performing surveys should improve response timing, all else being equal.

**Table 7:** Supply cost differences between standard surveys and surveys with and improved trap-and-lure combinations.

Species	Situation	Traps in improved grid (no.)	Supply cost difference^a^ ($)	Calculated trap+lure cost^b^ ($)	Default trap +lure cost ($)	Proportional difference in costs
Mexfly	Doubled attractiveness	317	$14,599	$49.16	$10.00	4.9
Navel orangeworm	Phero. vs. PPO+Phero.	382	$8,799	$23.03	$4.16	5.5
Tiger longicorn	Lure 1 vs. lure 3	112	$85,364	$762.18	$13.24	57.6
beetle	Likelihood ≈ 0.40	566	$41,038	$91.60	$17.94	5.1

These are for the proof-of-concept and case studies, with the equivalent cost of the improved trap-and-lure, the default cost, and the proportional difference between the two.

^a^ See Eqs. (2) and (3).

^b^ See Eq. (3).

Optimization of a survey probably does not always mean maximizing *p*(Capture), however, because with poorer traps and lures very high densities would be required^27^ and programs seem unlikely to have such large budgets. In the example of TLB, the best lure (#3) at a moderate trap density of 31.3 traps per km^2^ only achieved an estimated *p*(Capture) of 0.4 (Fig. 8). We think most managers would target *p*(Capture) values around 0.5^44^, but achieving that level of capture probability with the best current TLB lure might still be cost prohibitive. In some cases, modest additional spending could be worthwhile because it significantly increases *p*(Capture). For example, with NOW, further increasing the density from 2.3 traps per km^2^ (Fig. 7) to 3.5 per km^2^ (51% more) would cost an additional $4,600 overall but would increase *p*(Capture) from 0.43 to 0.56. The potential to reduce losses from pests^59^, if known, might be an additional factor to consider when choosing between survey plans, or considering increasing spending to achieve a greater *p*(Capture).

Over two decades ago, Mayo et al.^9^ demonstrated that servicing costs can be a much greater proportion of total costs in large-area surveys with longer durations. More recently, servicing costs have been singled out as a justification for developing remote monitoring methods e.g.,^60, 61^. That is because a system for identifying and counting targets electronically would eliminate repeated visits to the traps. In our results, mean servicing costs in enhanced surveys *after* trap-and-lure installation were about $9,200 for Mexfly, $5,700 for NOW, and about $3,800 for TLB. Saving those amounts would obviously be helpful. However, the imaging equipment for each trap and associated processing needs would also assuredly add to supply costs. Based on our results (Table 4), remote monitoring supply costs would need to be at least 55% less than default costs on average to be economical.

## Conclusion

Researchers understand some of the general cost implications of trapping surveys, such as that lower trap-and-lure densities and longer lure durations can reduce supply costs, or that remote monitoring could replace trap checks and associated travel, reducing servicing costs. However, most published research related to trap and lure costs has not quantitatively assessed survey costs. Basic supplies accounting can be insightful but understanding how different trap-and-lure options determine survey efficacy, and how that further affects design choices, should probably be done more often. Our results indicated that more attractive traps, when available, are worth using in most, if not all, surveys to reduce trap densities, since they contributed the most to overall costs. Greatly increased costs for improved traps-and-lures might challenge that but the noneconomic costs observed here were at least 5 times greater than base prices. In other cases, such as for increased lure durations, the savings were not as substantial. Still, combining thorough survey cost accounting with the quantification of survey efficacy demonstrated how trap-and-lure choices and densities could be “tuned” to provide an expected level of performance, rather relying on intuition, safety margins, “conservative” choices, or the like. Continued study of effective and economical survey methodologies will be critical for optimizing invasive pest management programs, especially for making them economically sustainable.

## Supplementary Information

Below is the link to the electronic supplementary material.

Supplementary Material 1

## Data Availability

No empirical data were collected for this study. All parameters and data generated or analyzed are included in this published article (and its Supplementary Information files).
