# Supplementary material for: Evaluating the impacts of trap and lure costs and attractiveness on invasive insect trapping designs
Source: Sci Rep. 2026 May 20;16:23052. doi: 10.1038/s41598-026-53691-1 (PMC13392245; doi:10.1038/s41598-026-53691-1)
Supplement: Supplementary file 1 — Supplementary Material 1 [file 41598_2026_53691_MOESM1_ESM.docx]

# Supplementary information for “Evaluating the Impacts of Trap and Lure Costs and Attractiveness on Invasive Insect Trapping Designs”

Lauren Stutts, Barney Caton, Hui Fang, Nicholas C. Manoukis, and Godshen Robert


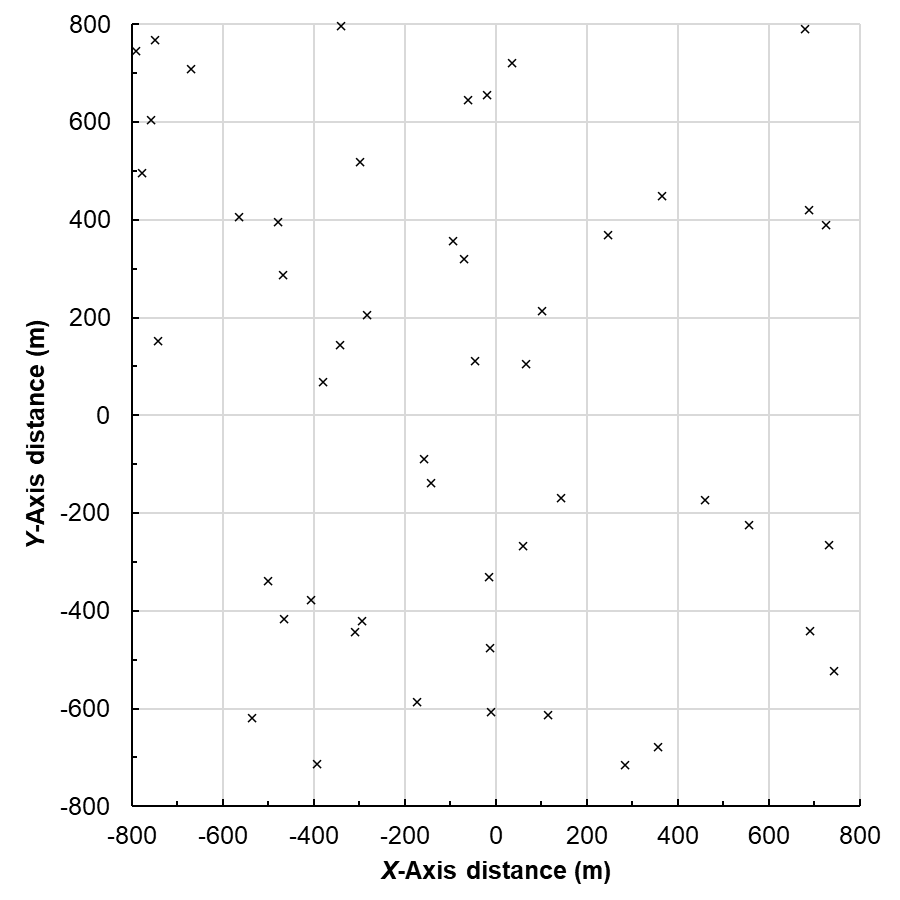


**Figure S1.** Locations of fifty random outbreak locations (Xs) in the central square mile of all simulations in TrapGrid.

**
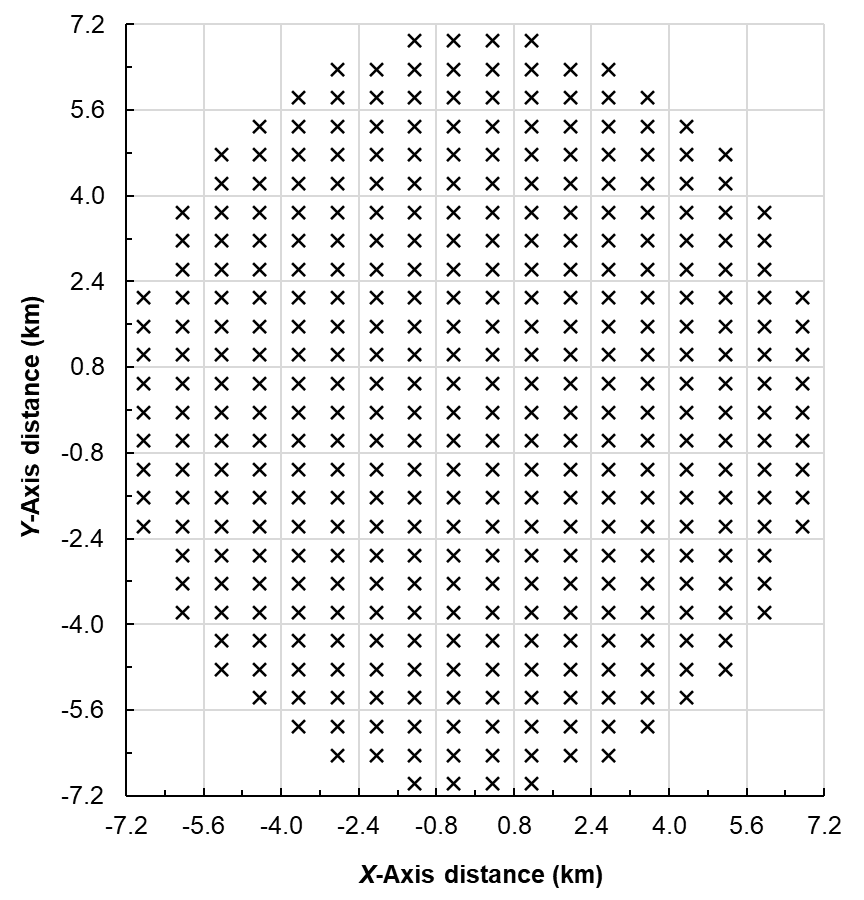
**

**Figure S2.** Locations of traps for delimitation of navel orangeworm at densities of 2.3 traps/km² (6 traps/mi²), for a total of 382 traps.


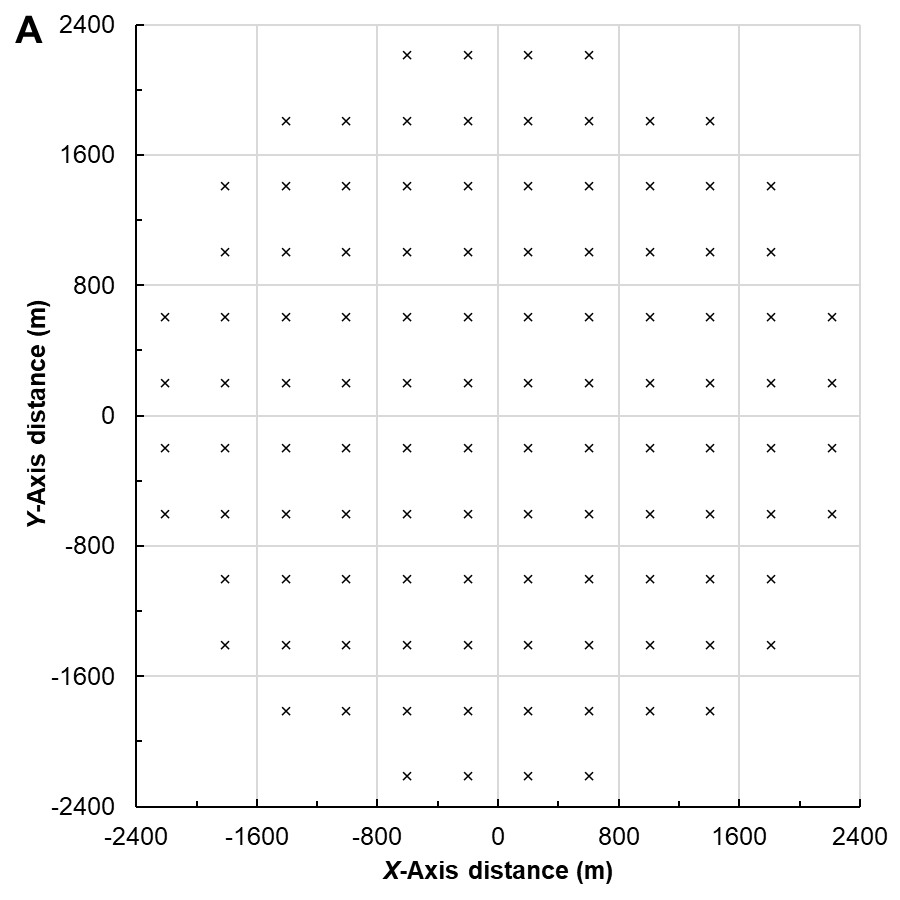

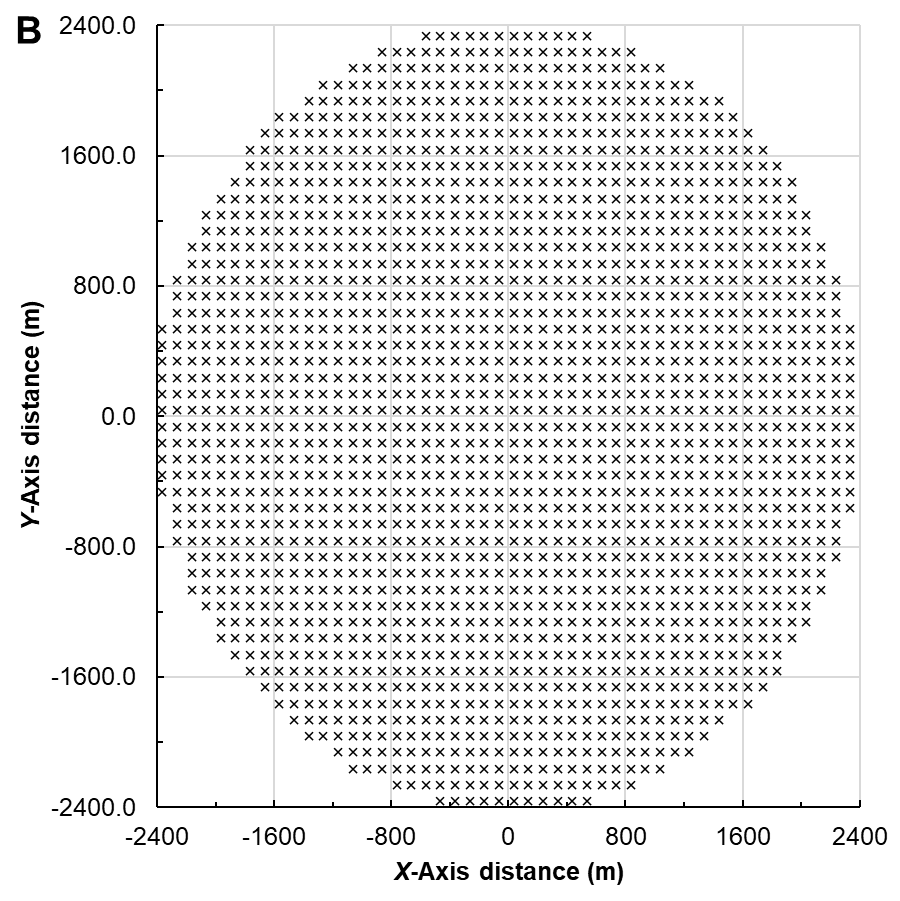


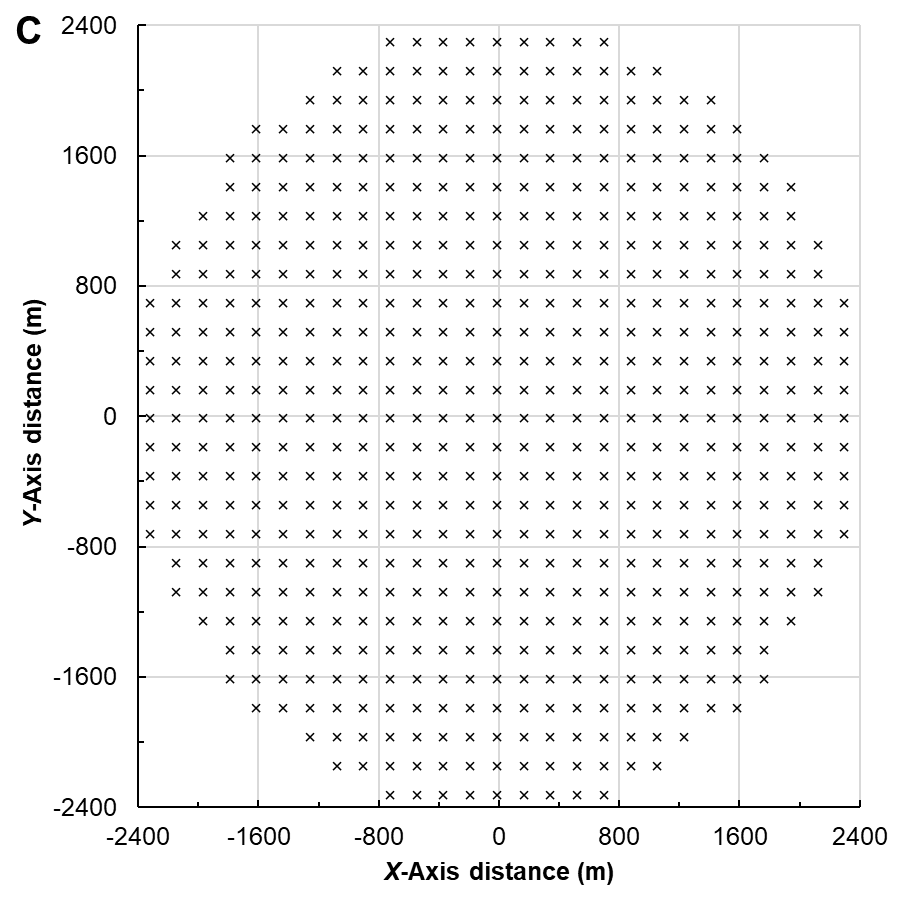


**Figure S3.** Locations of traps in grids with square-mile cells (2.6 km^2^) for delimitation of tiger longicorn beetle at densities of (A) 6.2 traps/km² (16 traps/mi²), (B) 98.8 traps/km² (256 traps/mi²), and (C) 31.3 traps/km² (81 traps/mi²).
